# From the microbiome to the central nervous system, an update on the epidemiology and pathogenesis of bacterial meningitis in childhood

**DOI:** 10.12688/f1000research.8533.1

**Published:** 2017-01-27

**Authors:** Andrew B Janowski, Jason G Newland

**Affiliations:** 1 Division of Pediatric Infectious Diseases, Washington University in St Louis, St. Louis, MO, USA

**Keywords:** meningitis, bacterial, vaccinations, risk factors

## Abstract

In the past century, advances in antibiotics and vaccination have dramatically altered the incidence and clinical outcomes of bacterial meningitis. We review the shifting epidemiology of meningitis in children, including after the implementation of vaccines that target common meningitic pathogens and the introduction of intrapartum antibiotic prophylaxis offered to mothers colonized with *Streptococcus agalactiae*. We also discuss what is currently known about the pathogenesis of meningitis. Recent studies of the human microbiome have illustrated dynamic relationships of bacterial and viral populations with the host, which may potentiate the risk of bacterial meningitis.

## Introduction

At the turn of the 20th century, bacterial meningitis was an almost universally fatal disease. Two important medical advances—antibiotics and vaccination—have dramatically decreased the incidence and the case fatality rate of bacterial meningitis, particularly within pediatric populations. Some of the pathogens that caused meningitis 20 years ago are now more likely to be encountered by medical trainees in reviewing textbooks than in clinical practice. With these shifting dynamics, a greater understanding of the current epidemiology of community-acquired meningitis is needed. In addition, several pathways involved in the pathogenesis of bacterial meningitis have been elucidated. We review some of these models and provide an update on the role of the microbiome in the development of meningitis.

## Overview of the epidemiology of bacterial meningitis in childhood

Traditional descriptions of bacterial meningitis in childhood have stratified causative pathogens on the basis of age, as there is a stark contrast in the bacterial pathogens that cause meningitis in newborns compared with older children. Meningitis in children older than 60 days, called “pediatric bacterial meningitis” in this review, is often caused by encapsulated bacteria that colonize the nasopharynx and other body sites. Meningitis in children younger than 60 days, called “young infant bacterial meningitis” in this review, is further stratified by gestational age and timing of onset of infection ^1^. In general, infections that occur within the first 7 days of life of a term neonate are described as early onset disease, whereas infections occurring from 7 to 60 days after birth are described as late-onset disease ^1^. Early onset disease is caused predominantly by bacteria transmitted at the time of parturition, whereas late-onset disease is caused by members of the microbiome transmitted at birth or through exposures after birth, such as maternal contact or method of feeding ^1– 5^. Despite the distinctions in pathogens between the age cohorts, pathogens of pediatric bacterial meningitis can also cause disease in young infants and vice versa.

## Pediatric bacterial meningitis

For over 30 years, the Centers for Disease Control and Prevention (CDC) in the US has published surveillance data of bacterial meningitis. In 1995, the CDC established the Active Bacterial Core Surveillance, an active monitoring system for invasive pathogens, and since then has made annual reports available to the public ( http://www.cdc.gov/abcs/reports-findings/surv-reports.html). The epidemiology of meningitis in the US has profoundly changed over the past several decades. In 1978–1981, *Haemophilus influenzae* was the most frequent cause of meningitis (48.3% of cases), followed by *Neisseria meningitidis* (19.6%) and *Streptococcus pneumoniae* (13.3%) ^6^. By 2014, in children under age 5 in the US, *S. pneumoniae* was the most frequently identified pathogen whereas *H. influenzae* was rarely detected ^7^.

**Figure 1.**
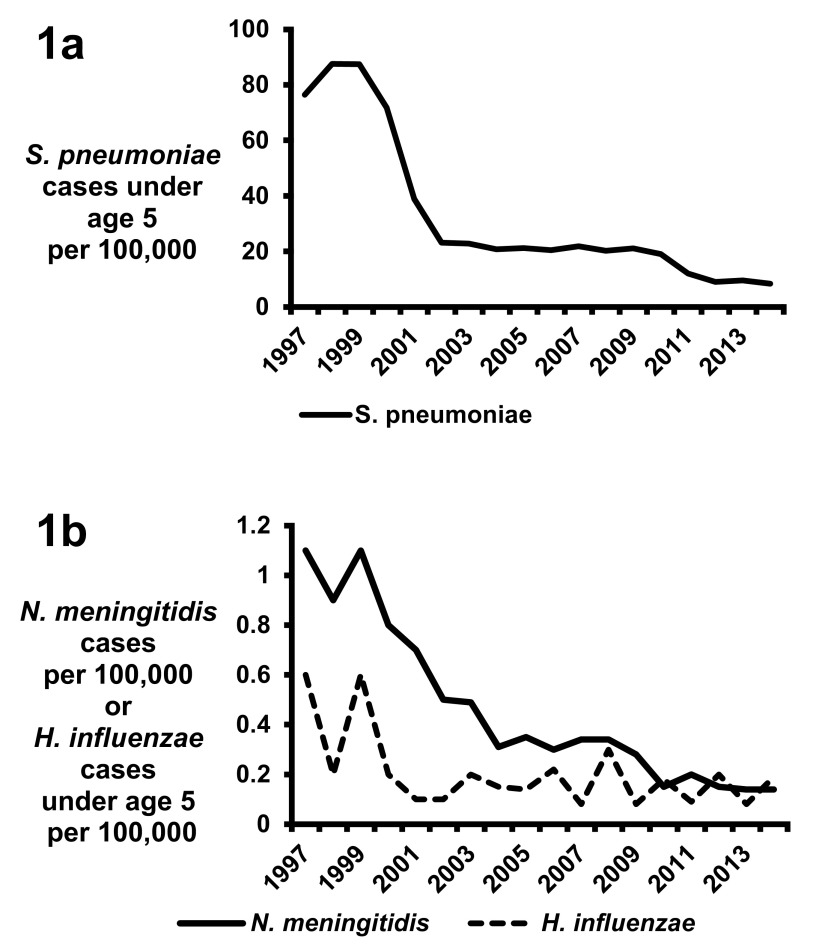
Rates of ( **a**) invasive disease for *Streptococcus pneumoniae* in children under the age of 5 and ( **b**) invasive disease caused by *Haemophilus influenzae* in children under the age of 5 and by *Neisseria meningitides* in all ages. All data are from accumulated Centers for Disease Control and Prevention (CDC) Active Bacterial Core surveillance reports 1997–2014 ( http://www.cdc.gov/abcs/reports-findings/surv-reports.html).

Although *S. pneumoniae* is the most frequent etiology of bacterial meningitis in the US, the incidence of pneumococcal meningitis has dramatically decreased over the past two decades because of the implementation of pneumococcal serotype vaccines ( Figure 1a) ^8^. In 2000, a seven-valent pneumococcal vaccine (PCV7) targeting a subset of serotypes associated with invasive disease was licensed in the US ^8^. After the introduction of PCV7, the rate of invasive disease caused by PCV7 serotypes fell from 80 per 100,000 population in 2000 to below one per 100,000 population in 2007 ^9^. Meningitis caused by PCV7 serotypes also significantly decreased, from 8.2 cases per 100,000 in 1998–1999 to 0.59 cases per 100,000 in 2004–2005 ^10^. Substantial reductions in invasive pneumococcal infections were also observed in other countries after implementation of the PCV7 vaccine ^11, 12^. However, surveillance data identified a rise in the incidence of meningitis caused by serotypes not included in the vaccine, known as serotype replacement ^13^, prompting the development of a 13-valent pneumococcal vaccine (PCV13) licensed in the US in 2010. Since the implementation of PCV13 in several countries, continued decreases in invasive *S. pneumoniae* diseases have been observed ( Figure 1a) ^14– 20^. However, there are conflicting data as to whether the introduction of PCV13 has decreased the rate of *S. pneumoniae* meningitis ^20– 22^.

The dramatic reduction in the incidence of *H. influenzae* meningitis demonstrates the tremendous success of the serotype B vaccine ^23^. *H. influenzae* type B is a highly virulent strain that caused the majority of *H. influenzae* meningitis cases ^23, 24^. In 1978–1981, the peak incidence of *H. influenzae* meningitis was in infants 9 to 11 months of age, and the attack rate was 70 cases of meningitis per 100,000 population per year ^6^. In the 1980s, several *H. influenzae* type B vaccines were in development; among them were an unconjugated polysaccharide formulation licensed in the US in 1985 and the conjugate vaccine licensed in the US in 1987. Implementation of the vaccines caused a rapid decline in *H. influenzae* disease; by 2014, the CDC identified only 40 invasive cases of *H. influenzae* type B infection, representing an invasive disease rate of 0.19 per 100,000 US children under the age of 5 ( Figure 1b) ^25– 27^. Similar analyses in other countries have also demonstrated a significant reduction of *H. influenzae* meningitis after implementation of the type B vaccine ^11, 12, 28^.

For *N. meningitidis*, 13 serogroups are currently known and only six serogroups are recognized to cause meningitis (A, B, C, W-135, X, and Y) ^29^. During 1978–1981 in the US, the highest rate of *N. meningitidis*-associated meningitis was in children aged 3 to 5 months, with over 10 cases per 100,000 population per year ^6^. Over the next 25 years, the incidence of meningitis caused by *N. meningitidis* decreased in the US and this was hypothesized to be due to a combination of environmental, organism, and host factors ( Figure 1b) ^30^. In 2005, the meningococcal conjugate quadrivalent vaccine (MenACWY) targeting serogroups A, C, Y, and W-135 was licensed for use in adolescents in the US. Further reductions in disease have been observed after implementation of the vaccine; in 2014, meningococcemia occurred in 0.14 cases per 100,000 persons in the US, representing a total of 443 invasive cases ^27^. Infants under a year of age have the highest incidence of meningitis from *N. meningitidis*; an estimated 2.74 cases of meningitis per 100,000 occurred in the US from 2006 to 2012 ^31^. From that same study, serogroup B caused 64% of cases of meningitis, serogroup Y caused 16%, and serogroup C caused 12% ^31^. Similar reductions in *N. meningitidis*-associated diseases have been observed in other countries after the implementation of vaccination strategies ^12, 32, 33^. *N. meningitidis* is a well-known cause of meningitis epidemics in the sub-Saharan region of Africa, where attack rates are as high as 800 cases per 100,000 population ^34, 35^. Serogroup A accounts for 80–85% of all outbreak cases, and many global efforts in distributing vaccines to epidemic regions of Africa have significantly reduced the incidence of meningitis ^35, 36^.

## Young infant bacterial meningitis

In older studies, *Streptococcus agalactiae* (Group B Streptococcus, or GBS) was the most frequently identified pathogen from cases of young infant bacterial meningitis, followed in incidence by other organisms, including *Escherichia coli* and *Listeria monocytogenes* ^37– 42^. In a 2014 study from the UK and Ireland, GBS remained the most common cause of meningitis despite interventions to reduce disease caused by this organism ^43^. This result contrasts with a 2014 study of young infants in California, where *E. coli* was the most frequently identified pathogen in meningitis ^44^. Other recent studies have described the importance of enteric Gram-negative organisms causing meningitis in this age group, while the incidence of meningitis caused by *L. monocytogenes* has decreased ^44– 46^.

**Figure 2.**
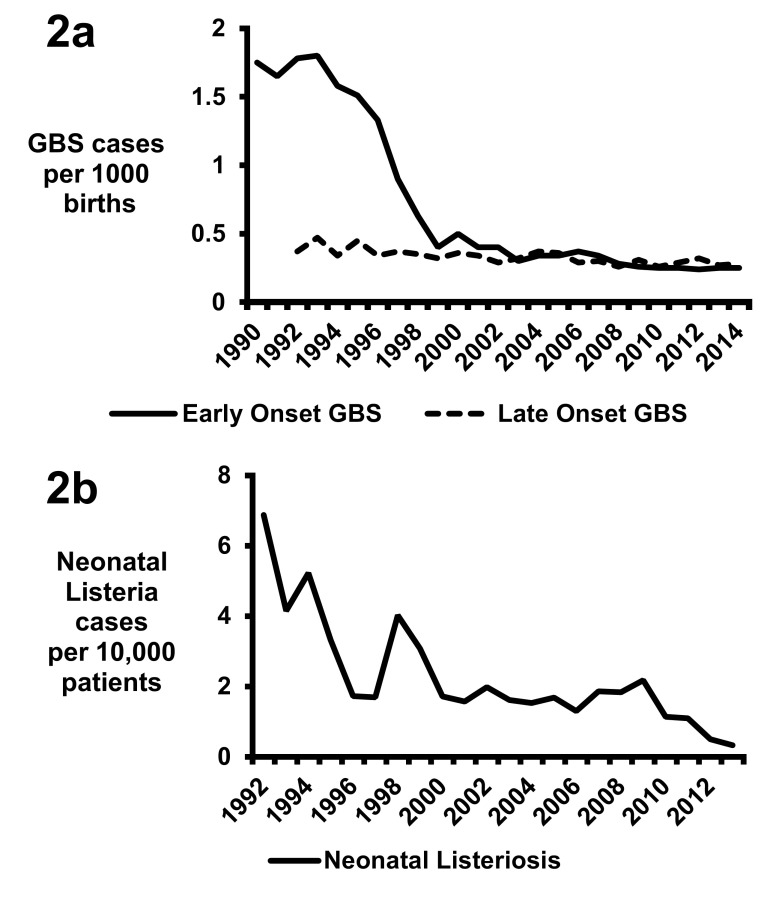
Rates of invasive young infant infections in the United States. ( **a**) Early onset Group B Streptococcus (GBS) disease data for 1990 to 1998 are from 47 and for 1999 to 2014 are from accumulated Centers for Disease Control and Prevention (CDC) Active Bacterial Core surveillance reports. Late-onset GBS disease data for 1992–2005 are from 59 and for 2006–2014 are from accumulated CDC Active Bacterial Core surveillance reports. ( **b**) Listeria data are from 82.

The primary reason behind the shift in the epidemiology in the US has been the implementation of intrapartum antibiotic prophylaxis against GBS ^39, 47, 48^. The CDC, the American Academy of Pediatrics, and the American College of Obstetricians and Gynecologists published unified prophylaxis guidelines in 1996, screening guidelines in 2002, and revised guidelines in 2010 ^49– 55^. Pregnant mothers are screened for rectovaginal colonization at 35 to 37 weeks’ gestation for GBS, and colonized mothers are provided with intrapartum antibiotic prophylaxis ^52– 54^. Additionally, intrapartum antibiotics are indicated for mothers if they have a previous infant with invasive GBS disease, history of GBS bacteriuria, or unknown GBS status with at least one of the following: delivery at less than 37 weeks’ gestation, amniotic membrane rupture of at least 18 hours, fever, or an intrapartum nucleic acid amplification test (NAAT) positive for GBS ^55^. Efficacy of reducing transmission is enhanced if a beta-lactam or cephalosporin antibiotic is given at least 4 hours prior to delivery ^56, 57^. This intervention has dramatically reduced the rates of early onset sepsis from GBS, including meningitis ( Figure 2a) ^48^. However, intrapartum prophylaxis has not reduced the rate of late-onset GBS invasive disease ( Figure 2a) ^48, 58, 59^. Multiple factors likely contribute to the unchanged incidence of GBS late-onset disease, as intrapartum antibiotics reduce but do not abrogate GBS colonization, and transmission of GBS after birth may also occur through maternal, nosocomial, or environmental contacts ^3, 59^.

The overall incidence of meningitis and sepsis from *E. coli* has remained relatively stable in term infants since the implementation of GBS prophylaxis guidelines in the US and France ^60– 64^. However, trends of increasing frequency of disease in select subgroups have been described, including an increase of *E. coli* early onset sepsis in preterm or very-low-birth-weight neonates ^1, 64– 66^. The incidence of late-onset disease from *E. coli* has also increased in term or preterm infants ^1, 64– 66^. *E. coli* isolated from cases of meningitis is frequently resistant to ampicillin ^45, 60^, but an increase in ampicillin-resistant *E. coli* has been observed only in low-birth-weight or premature infants ^60, 67– 69^. Recent analyses of the infant gastrointestinal microbiome have identified the presence of many antibiotic-resistance genes, but it is not known why the frequency of resistant *E. coli* invasive disease has not increased in all young infants during the period of intrapartum prophylaxis ^70– 74^.

Previously in the US, *L. monocytogenes* was a common cause of neonatal meningitis, but in 2014 only 13 cases of meningitis and 37 cases of bacteremia were reported in neonates ^75^. Based on the birth data for the US in 2014 (3,988,076 total births), the 50 cases of neonatal listeriosis would translate to a rate of 1.25 invasive cases per 100,000 births. This is in stark contrast to 17.4 invasive cases per 100,000 births in 1989, which decreased to 8.6 cases per 100,000 births by 1993 ^76^. Increased safety in food product handling was the major driving force in the reduction of cases in the US during the 1980s and 1990s ^77– 79^. By 2004–2009, seven cases per 100,000 births on average were complicated by *L. monocytogenes* infection, according to estimates calculated by using data from Silk *et al*. ^80^ and the US birth rate ^81^. The GBS intrapartum prophylaxis recommendations may have contributed to the reduction of listeriosis, as the primary antibiotics used for prophylaxis—penicillin G and ampicillin—have excellent activity against *L. monocytogenes* ^82^. Supporting this hypothesis are reduced rates of invasive disease in infants under 30 days of life identified from the Pediatric Health Information System, a database that uses pediatric discharge data from 45 tertiary pediatric hospitals in the US ^82^. A total of 6.87 listeria cases per 10,000 patients occurred in 1992 compared with 0.33 cases per 10,000 patients in 2013, and this correlated with the reduction in GBS invasive diseases ( Figure 2b) ^82^.

## Pathogenesis of meningitis

**Figure 3.**
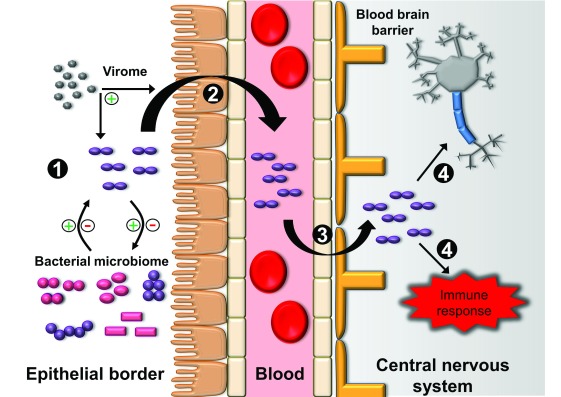
Four generalized steps are involved in the pathogenesis of bacterial meningitis. ( **1**) Bacterial colonization of the epithelial border. Colonization is affected by the host and other members of the microbiome, including bacteria and viruses (virome). Bacteria may have synergistic or antagonistic effects on colonization, while viruses may enhance colonization. ( **2**) Bacterial invasion of the epithelial surface into the bloodstream. This process can be enhanced by viruses. ( **3**) Bacterial breeching of the blood-brain barrier. Various pathways have been described in the penetration of the blood-brain barrier, including transcellular, paracellular, and “Trojan horse” mechanisms of entry. ( **4**) Bacterial replication in the central nervous system. The release of bacterial products causes direct toxicity to neurons and stimulation of the immune response, which contributes to additional neurotoxicity.

The pathogenesis of bacterial meningitis is often characterized by four primary processes: (1) colonization of the epithelial barrier, (2) entrance into the circulatory system, (3) breeching of the blood-brain barrier (BBB), and (4) central nervous system (CNS) inflammation and injury ( Figure 3) ^83– 86^.

Epithelial surfaces in humans are the interface in which complex interactions develop among the host, environment, and diverse populations of organisms. *S. pneumoniae*, *H. influenzae*, *N. meningitidis*, and many other bacterial organisms colonize the nasopharyngeal tract, being freely exchanged by aerosolization and contact with secretions ^87, 88^. Although these organisms collectively inhabit the nasopharynx, this colonization is not necessarily a peaceful co-inhabitation between organisms; rather it is an evolving balance among mutualism, competition, and outright antagonism. *S. pneumoniae* can produce hydrogen peroxide that causes a rapid decrease in the growth of *H. influenzae* ^89^. Likewise, *H. influenzae* type B can induce an immune response that selectively targets *S. pneumoniae* while leaving *H. influenzae* colonies unscathed ^90, 91^. Vaccination efforts have also altered the composition of the nasopharyngeal microbiome and have altered the epidemiology of acute otitis media ^92, 93^. Ultimately, there are many inter-bacterial interactions in the nasopharynx, and further investigation may reveal compositions of the microbiome that modify the risk of meningitis ^92, 94, 95^.

Interactions with the host are not limited to the bacterial domain, as co-inhabitation of specific viruses with bacteria led to synergistic relationships ^88^. Viruses can contribute to bacterial adherence to epithelial surfaces through viral factors and upregulation of host adhesion proteins ^88^. Viruses also can contribute to the bacterial invasion of the epithelial surfaces by causing disruption of the epithelial barrier and by impairing the immune response ^88, 96– 98^. Associations between viral infection and meningitis have been observed, and these associations may partially explain outbreaks or seasonality of meningitis ^99– 103^.

Most of the bacterial pathogens of young infant and pediatric meningitis contain a polysaccharide capsule that contributes to invasion of the epithelial surface and survival in the bloodstream ^48, 84, 104– 109^. The capsule is an important virulence factor that confers added protection from phagocytosis, complement pathways, and penetration of the epithelium and BBB ^48, 84, 85, 110– 112^. Children with antibody deficiencies, defects of the complement pathway, or asplenia are at particular risk from invasive disease of these pathogens because of their diminished ability to target and clear encapsulated pathogens from the bloodstream ^113^.

Bacterial pathogens most often reach the BBB via the bloodstream. A threshold of bacteremia contributes to the breeching of the BBB, as a higher quantity of bacteria in the bloodstream is associated with increased risk of developing meningitis ^104, 114– 117^. Various mechanisms of bacterial factors have been established in the penetration of the BBB. The capsule can aid in bacterial transcellular crossing of the BBB, along with other attachment proteins ^83– 85, 104, 118^. Other mechanisms of crossing the BBB, including paracellular pathways or the “Trojan horse” mechanism of infected phagocytes, have been identified ^83– 85, 104^. Meningitis can also occur through direct compromise of the BBB, and mechanisms include penetrating injuries, congenital defects, adjacent infections with erosion into the CNS, or neurosurgical procedures ^119– 125^.

Upon entry of bacterial pathogens into the CNS, they rapidly divide, as the CNS is devoid of complement, antibodies, and opsonic proteins ^85, 86^. The immune response is activated by Toll-like receptors and Nod-like receptors recognizing pathogen-associated molecular patterns ^126, 127^. These signaling pathways lead to the production of proinflammatory cytokines and mobilization of the immune response, leading to pleocytosis of white blood cells ^83– 86, 126^. Bacterial cell wall material, enzymes, and toxins cause direct injury to neurons and indirect damage by increasing vascular permeability that causes edema and further injury ^83– 86^. Neuronal injury is also caused by toxic molecules released by the immune response, including reactive oxygen species, nitrous oxide, and peroxynitrite ^84– 86^. The release of proteases and excitatory amino acids by the immune response also contributes to neurotoxicity ^84– 86^.

## Special considerations of young infant meningitis

Inoculation of bacteria into mucosal surfaces occurs prior to and during parturition with subsequent bacterial invasion causing early onset sepsis ^48^. Late-onset sepsis is associated with a period of asymptomatic bacterial colonization and subsequent invasion ^3– 5^. Two sites of colonization likely contribute to cases of late-onset meningitis. The gastrointestinal tract serves as a frequent site of colonization for *E. coli*, GBS, and *L. monocytogenes*, and all of these pathogens have essential factors that allow for epithelial adherence and invasion ^48, 85, 128^. GBS is readily transmitted from mother to neonate; 29–85% (mean rate of approximately 50%) of infants born to a GBS-positive mother become colonized ^48^. The second potential site of colonization is the urinary tract, which may harbor asymptomatic bacteriuria ^129, 130^. Ascending infection can lead to seeding of the kidney, bacteremia, and then meningitis, as around 13.2% of febrile young infants will present with a urinary tract infection, and a smaller subset will have simultaneous evidence of bacteriuria, bacteremia, and meningitis ^44, 131^.

In premature infants, bacteremic events can be preceded by colonization of the gut by the causative pathogen ^132^. In a prospective monitoring of stool samples, Carl *et al*. captured seven cases of sepsis in which the causative bacterial pathogen was also identified from the patient’s stool sample preceding the episode of bacteremia ^132^. It is unclear whether similar events occur in term infants or within other body sites that contribute to invasion of these bacterial organisms. The effects of intrapartum antibiotics on the infant microbiome are also being elucidated, as the gastrointestinal microbiome of infants born to mothers who received intrapartum antibiotics is different from that of infants born to untreated mothers ^133– 136^. However, the consequences, if any, of these microbial communities for the risk of meningitis are unknown.

Recent analysis of the microbiome has shown dynamic colonization of the neonatal gut, and one potential factor in colonization is the population of bacteriophages ^137, 138^. In a longitudinal study of healthy twins, the neonatal bacterial microbiome gained bacterial diversity with increased age, and conversely bacteriophage diversity decreased with age ^137^. This may suggest an essential relationship between bacteriophages and development of the gut bacterial microbiome, in which the bacteriophage population guides the diversity of the bacterial population. Further data are needed to determine whether the risk of invasive bacterial disease is increased by certain compositions of the microbiome and whether bacteriophage populations potentiate this risk ^139^.

The relatively immature immune system of the neonate also contributes to the invasive risk of bacterial pathogens ^140– 142^. Defects in phagocytic cell function, chemotaxis, cytokine production, complement pathways, Toll-like receptor responses, and antibody production are further conducive to invasive disease ^140– 142^. These defects also include adaptive immunity in response to viral infections, including lymphocyte proliferation and antibody responses ^142– 144^. Though significant, these immune defects are transient, likely contributing to the decreased incidence of serious bacterial infections with increasing age.

## Conclusions

The incidence of bacterial meningitis in children has been dramatically reduced and this is primarily because of immunization and intrapartum prophylaxis strategies. Nonetheless, *E. coli*, GBS, *S. pneumoniae*, and *N. meningitidis* remain important pathogens of meningitis. Recent advances in the analysis of the microbiome have expanded the understanding of the pathogenesis of meningitis. These new insights will provide new avenues of research and may stimulate the development of future treatments to prevent and treat meningitis.

## References

[1] Simonsen KA Anderson-Berry AL Delair SF: Early-onset neonatal sepsis. *Clin Microbiol Rev.* 2014;27(1):21–47. 10.1128/CMR.00031-13 24396135 PMC3910904

[2] Le Doare K Kampmann B: Breast milk and Group B streptococcal infection: vector of transmission or vehicle for protection? *Vaccine.* 2014;32(26):3128–32. 10.1016/j.vaccine.2014.04.020 24736004 PMC4037808

[3] Berardi A Cattelani C Creti R: Group B streptococcal infections in the newborn infant and the potential value of maternal vaccination. *Expert Rev Anti Infect Ther.* 2015;13(11):1387–99. 10.1586/14787210.2015.1079126 26295167

[4] Dong Y Speer CP: Late-onset neonatal sepsis: recent developments. *Arch Dis Child Fetal Neonatal Ed.* 2015;100(3):F257–63. 10.1136/archdischild-2014-306213 25425653 PMC4413803

[5] Barichello T Fagundes GD Generoso JS: Pathophysiology of neonatal acute bacterial meningitis. *J Med Microbiol.* 2013;62(Pt 12):1781–9. 10.1099/jmm.0.059840-0 23946474

[6] Schlech WF 3rd Ward JI Band JD: Bacterial meningitis in the United States, 1978 through 1981. The National Bacterial Meningitis Surveillance Study. *JAMA.* 1985;253(12):1749–54. 10.1001/jama.1985.03350360075022 3871869

[7] Centers for Disease Control and Prevention: Active Bacterial Core Surveillance Report, Emerging Infections Program Network, *Streptococcus pneumoniae*, 2014.2014 Reference Source

[8] Nuorti JP Whitney CG Centers for Disease Control and Prevention (CDC): Prevention of pneumococcal disease among infants and children - use of 13-valent pneumococcal conjugate vaccine and 23-valent pneumococcal polysaccharide vaccine - recommendations of the Advisory Committee on Immunization Practices (ACIP). *MMWR Recomm Rep.* 2010;59(RR-11):1–18. 21150868

[9] Cox CM Link-Gelles R: Chapter 11: Pneumococcal. In *Manual for the surveillance of vaccine-preventable diseases* (ed), Dept. of Health and Human Services Centers for Disease Control and Prevention, Atlanta, Ga.2012 Reference Source

[10] Hsu HE Shutt KA Moore MR: Effect of pneumococcal conjugate vaccine on pneumococcal meningitis. *N Engl J Med.* 2009;360(3):244–56. 10.1056/NEJMoa0800836 19144940 PMC4663990

[11] Brouwer MC Tunkel AR van de Beek D: Epidemiology, diagnosis, and antimicrobial treatment of acute bacterial meningitis. *Clin Microbiol Rev.* 2010;23(3):467–92. 10.1128/CMR.00070-09 20610819 PMC2901656

[12] McIntyre PB O'Brien KL Greenwood B: Effect of vaccines on bacterial meningitis worldwide. *Lancet.* 2012;380(9854):1703–11. 10.1016/S0140-6736(12)61187-8 23141619

[13] Weinberger DM Malley R Lipsitch M: Serotype replacement in disease after pneumococcal vaccination. *Lancet.* 2011;378(9807):1962–73. 10.1016/S0140-6736(10)62225-8 21492929 PMC3256741

[14] Moore MR Link-Gelles R Schaffner W: Effect of use of 13-valent pneumococcal conjugate vaccine in children on invasive pneumococcal disease in children and adults in the USA: analysis of multisite, population-based surveillance. *Lancet Infect Dis.* 2015;15(3):301–9. 10.1016/S1473-3099(14)71081-3 25656600 PMC4876855

[15] Levy C Varon E Picard C: Trends of pneumococcal meningitis in children after introduction of the 13-valent pneumococcal conjugate vaccine in France. *Pediatr Infect Dis J.* 2014;33(12):1216–21. 10.1097/INF.0000000000000451 25037044

[16] Andrews NJ Waight PA Burbidge P: Serotype-specific effectiveness and correlates of protection for the 13-valent pneumococcal conjugate vaccine: a postlicensure indirect cohort study. *Lancet Infect Dis.* 2014;14(9):839–46. 10.1016/S1473-3099(14)70822-9 25042756

[17] Ben-Shimol S Greenberg D Givon-Lavi N: Early impact of sequential introduction of 7-valent and 13-valent pneumococcal conjugate vaccine on IPD in Israeli children <5 years: an active prospective nationwide surveillance. *Vaccine.* 2014;32(27):3452–9. 10.1016/j.vaccine.2014.03.065 24690148

[18] Demczuk WH Martin I Griffith A: Serotype distribution of invasive *Streptococcus pneumoniae* in Canada after the introduction of the 13-valent pneumococcal conjugate vaccine, 2010–2012. *Can J Microbiol.* 2013;59(12):778–88. 10.1139/cjm-2013-0614 24313450

[19] Harboe ZB Dalby T Weinberger DM: Impact of 13-valent pneumococcal conjugate vaccination in invasive pneumococcal disease incidence and mortality. *Clin Infect Dis.* 2014;59(8):1066–73. 10.1093/cid/ciu524 25034421

[20] Guevara M Ezpeleta C Gil-Setas A: Reduced incidence of invasive pneumococcal disease after introduction of the 13-valent conjugate vaccine in Navarre, Spain, 2001–2013. *Vaccine.* 2014;32(22):2553–62. 10.1016/j.vaccine.2014.03.054 24674661

[21] Olarte L Barson WJ Barson RM: Impact of the 13-Valent Pneumococcal Conjugate Vaccine on Pneumococcal Meningitis in US Children. *Clin Infect Dis.* 2015;61(5):767–75. 10.1093/cid/civ368 25972022

[22] Cohen R Biscardi S Levy C: The multifaceted impact of pneumococcal conjugate vaccine implementation in children in France between 2001 to 2014. *Hum Vaccin Immunother.* 2016;12(2):277–84. 10.1080/21645515.2015.1116654 26905678 PMC5049719

[23] Thigpen MC Whitney CG Messonnier NE: Bacterial meningitis in the United States, 1998–2007. *N Engl J Med.* 2011;364(21):2016–25. 10.1056/NEJMoa1005384 21612470

[24] Pittman M: Variation And Type Specificity In The Bacterial Species Hemophilus Influenzae. *J Exp Med.* 1931;53(4):471–92. 10.1084/jem.53.4.471 19869858 PMC2131978

[25] Adams D Fullerton K Jajosky R: Summary of Notifiable Infectious Diseases and Conditions - United States, 2013. *MMWR Morb Mortal Wkly Rep.* 2015;62(53):1–122. 10.15585/mmwr.mm6253a1 26492038

[26] Centers for Disease Control and Prevention: Active Bacterial Core Surveillance Report, Emerging Infections Program Network, *Haemophilus influenza* 2014.2014 Reference Source

[27] Adams DA Thomas KR Jajosky RA: Summary of Notifiable Infectious Diseases and Conditions - United States, 2014. *MMWR Morb Mortal Wkly Rep.* 2016;63(54):1–152. 10.15585/mmwr.mm6354a1 27736829

[28] Peltola H: Worldwide *Haemophilus influenzae* type b disease at the beginning of the 21st century: global analysis of the disease burden 25 years after the use of the polysaccharide vaccine and a decade after the advent of conjugates. *Clin Microbiol Rev.* 2000;13(2):302–17. 10.1128/CMR.13.2.302-317.2000 10756001 PMC100154

[29] Rouphael NG Stephens DS: *Neisseria meningitidis*: biology, microbiology, and epidemiology. *Methods Mol Biol.* 2012;799:1–20. 10.1007/978-1-61779-346-2_1 21993636 PMC4349422

[30] Cohn AC MacNeil JR Harrison LH: Changes in *Neisseria meningitidis* disease epidemiology in the United States, 1998–2007: implications for prevention of meningococcal disease. *Clin Infect Dis.* 2010;50(2):184–91. 10.1086/649209 20001736

[31] MacNeil JR Bennett N Farley MM: Epidemiology of infant meningococcal disease in the United States, 2006–2012. *Pediatrics.* 2015;135(2):e305–11. 10.1542/peds.2014-2035 25583921 PMC4803024

[32] Sáfadi MA McIntosh ED: Epidemiology and prevention of meningococcal disease: a critical appraisal of vaccine policies. *Expert Rev Vaccines.* 2011;10(12):1717–30. 10.1586/erv.11.159 22085175

[33] Borrow R Alarcón P Carlos J: The Global Meningococcal Initiative: global epidemiology, the impact of vaccines on meningococcal disease and the importance of herd protection. *Expert Rev Vaccines.* 2016:1–16. 10.1080/14760584.2017.1258308 27820969

[34] Scarborough M Thwaites GE: The diagnosis and management of acute bacterial meningitis in resource-poor settings. *Lancet Neurol.* 2008;7(7):637–48. 10.1016/S1474-4422(08)70139-X 18565457

[35] Cohn A MacNeil J: The Changing Epidemiology of Meningococcal Disease. *Infect Dis Clin North Am.* 2015;29(4):667–77. 10.1016/j.idc.2015.08.002 26610420

[36] Collard J Issaka B Zaneidou M: Epidemiological changes in meningococcal meningitis in Niger from 2008 to 2011 and the impact of vaccination. *BMC Infect Dis.* 2013;13:576. 10.1186/1471-2334-13-576 24313998 PMC4029580

[37] Gladstone IM Ehrenkranz RA Edberg SC: A ten-year review of neonatal sepsis and comparison with the previous fifty-year experience. *Pediatr Infect Dis J.* 1990;9(11):819–25. 10.1097/00006454-199011000-00009 2263432

[38] Wiswell TE Baumgart S Gannon CM: No lumbar puncture in the evaluation for early neonatal sepsis: will meningitis be missed? *Pediatrics.* 1995;95(6):803–6. 7761203

[39] Camacho-Gonzalez A Spearman PW Stoll BJ: Neonatal infectious diseases: evaluation of neonatal sepsis. *Pediatr Clin North Am.* 2013;60(6):367–89. 10.1016/j.pcl.2012.12.003 23481106 PMC4405627

[40] Gaschignard J Levy C Romain O: Neonatal Bacterial Meningitis: 444 Cases in 7 Years. *Pediatr Infect Dis J.* 2011;30(3):212–7. 10.1097/INF.0b013e3181fab1e7 21416693

[41] Chang C Chang WN Huang LT: Bacterial meningitis in infants: the epidemiology, clinical features, and prognostic factors. *Brain Dev.* 2004;26(3):168–75. 10.1016/S0387-7604(03)00122-0 15030905

[42] Edmond KM Kortsalioudaki C Scott S: Group B streptococcal disease in infants aged younger than 3 months: systematic review and meta-analysis. *Lancet.* 2012;379(9815):547–56. 10.1016/S0140-6736(11)61651-6 22226047

[43] Okike IO Johnson AP Henderson KL: Incidence, etiology, and outcome of bacterial meningitis in infants aged <90 days in the United kingdom and Republic of Ireland: prospective, enhanced, national population-based surveillance. *Clin Infect Dis.* 2014;59(10):e150–7. 10.1093/cid/ciu514 24997051

[44] Greenhow TL Hung YY Herz AM: The changing epidemiology of serious bacterial infections in young infants. *Pediatr Infect Dis J.* 2014;33(6):595–9. 10.1097/INF.0000000000000225 24326416

[45] Byington CL Rittichier KK Bassett KE: Serious bacterial infections in febrile infants younger than 90 days of age: the importance of ampicillin-resistant pathogens. *Pediatrics.* 2003;111(5 Pt 1):964–8. 10.1542/peds.111.5.964 12728072

[46] Sadow KB Derr R Teach SJ: Bacterial infections in infants 60 days and younger: epidemiology, resistance, and implications for treatment. *Arch Pediatr Adolesc Med.* 1999;153(6):611–4. 10.1001/archpedi.153.6.611 10357302

[47] Schrag SJ Zywicki S Farley MM: Group B streptococcal disease in the era of intrapartum antibiotic prophylaxis. *N Engl J Med.* 2000;342(1):15–20. 10.1056/NEJM200001063420103 10620644

[48] Edwards MS Nizet V Baker CJ: Group B Streptococcal Infections. In *Remington and Klein's infectious diseases of the fetus and newborn infant*, 8th ed. Elsevier/Saunders, Philadelphia.2016 Reference Source

[49] Prevention of perinatal group B streptococcal disease: a public health perspective. Centers for Disease Control and Prevention. *MMWR Recomm Rep.* 1996;45(RR-7):1–24. 8637497

[50] ACOG committee opinion. Prevention of early-onset group B streptococcal disease in newborns. Number 173--June 1996. Committee on Obstetric Practice. American College of Obstetrics and Gynecologists. *Int J Gynaecol Obstet.* 1996;54(2):197–205. 9236325

[51] Revised guidelines for prevention of early-onset group B streptococcal (GBS) infection. American Academy of Pediatrics Committee on Infectious Diseases and Committee on Fetus and Newborn. *Pediatrics.* 1997;99(3):489–96. 10.1542/peds.99.3.489 9041310

[52] Schrag S Gorwitz R Fultz-Butts K: Prevention of perinatal group B streptococcal disease. Revised guidelines from CDC. *MMWR Recomm Rep.* 2002;51(RR-11):1–22. 12211284

[53] American College of Obstetricians and Gynecologists: ACOG Committee Opinion: number 279, December 2002. Prevention of early-onset group B streptococcal disease in newborns. *Obstet Gynecol.* 2002;100(6):1405–12. 10.1016/S0029-7844(02)02629-7 12468196

[54] Committee on Infectious Diseases, American Academy of Pediatrics, Kimberlin DW BMT, : Group B Streptococcal infections in Red book 2003 : report of the Committee on Infectious Diseases. 26. ed. American Academy of Pediatrics; BMJ, Washington, D.C. London.2003;927 Reference Source

[55] Verani JR McGee L Schrag SJ: Prevention of perinatal group B streptococcal disease--revised guidelines from CDC, 2010. *MMWR Recomm Rep.* 2010;59(RR-10):1–36. 21088663

[56] de Cueto M Sanchez MJ Sampedro A: Timing of intrapartum ampicillin and prevention of vertical transmission of group B streptococcus. *Obstet Gynecol.* 1998;91(1):112–4. 10.1016/S0029-7844(97)00578-4 9464732

[57] Turrentine MA Greisinger AJ Brown KS: Duration of intrapartum antibiotics for group B streptococcus on the diagnosis of clinical neonatal sepsis. *Infect Dis Obstet Gynecol.* 2013;2013: 525878. 10.1155/2013/525878 23606801 PMC3625608

[58] Phares CR Lynfield R Farley MM: Epidemiology of invasive group B streptococcal disease in the United States, 1999–2005. *JAMA.* 2008;299(17):2056–65. 10.1001/jama.299.17.2056 18460666

[59] Jordan HT Farley MM Craig A: Revisiting the need for vaccine prevention of late-onset neonatal group B streptococcal disease: a multistate, population-based analysis. *Pediatr Infect Dis J.* 2008;27(12):1057–64. 10.1097/INF.0b013e318180b3b9 18989238

[60] Basmaci R Bonacorsi S Bidet P: *Escherichia Coli* Meningitis Features in 325 Children From 2001 to 2013 in France. *Clin Infect Dis.* 2015;61(5):779–86. 10.1093/cid/civ367 25944342

[61] Baltimore RS Huie SM Meek JI: Early-onset neonatal sepsis in the era of group B streptococcal prevention. *Pediatrics.* 2001;108(5):1094–8. 10.1542/peds.108.5.1094 11694686

[62] Edwards RK Jamie WE Sterner D: Intrapartum antibiotic prophylaxis and early-onset neonatal sepsis patterns. *Infect Dis Obstet Gynecol.* 2003;11(4):221–6. 10.1080/10647440300025525 15108869 PMC1852291

[63] Daley AJ Isaacs D Australasian Study Group for Neonatal Infections: Ten-year study on the effect of intrapartum antibiotic prophylaxis on early onset group B streptococcal and Escherichia coli neonatal sepsis in Australasia. *Pediatr Infect Dis J.* 2004;23(7):630–4. 10.1097/01.inf.0000128782.20060.79 15247601

[64] Bauserman MS Laughon MM Hornik CP: Group B Streptococcus and Escherichia coli infections in the intensive care nursery in the era of intrapartum antibiotic prophylaxis. *Pediatr Infect Dis J.* 2013;32(3):208–12. 23011013 10.1097/INF.0b013e318275058a PMC3572304

[65] Bizzarro MJ Dembry L Baltimore RS: Changing patterns in neonatal *Escherichia coli* sepsis and ampicillin resistance in the era of intrapartum antibiotic prophylaxis. *Pediatrics.* 2008;121(4):689–96. 10.1542/peds.2007-2171 18381532

[66] Stoll BJ Hansen N Fanaroff AA: Changes in pathogens causing early-onset sepsis in very-low-birth-weight infants. *N Engl J Med.* 2002;347(4):240–7. 10.1056/NEJMoa012657 12140299

[67] Schrag SJ Hadler JL Arnold KE: Risk factors for invasive, early-onset Escherichia coli infections in the era of widespread intrapartum antibiotic use. *Pediatrics.* 2006;118(2):570–6. 10.1542/peds.2005-3083 16882809

[68] Moore MR Schrag SJ Schuchat A: Effects of intrapartum antimicrobial prophylaxis for prevention of group-B-streptococcal disease on the incidence and ecology of early-onset neonatal sepsis. *Lancet Infect Dis.* 2003;3(4):201–13. 10.1016/S1473-3099(03)00577-2 12679263

[69] Puopolo KM Eichenwald EC: No change in the incidence of ampicillin-resistant, neonatal, early-onset sepsis over 18 years. *Pediatrics.* 2010;125(5):e1031–8. 10.1542/peds.2009-1573 20385650

[70] Moore AM Ahmadi S Patel S: Gut resistome development in healthy twin pairs in the first year of life. *Microbiome.* 2015;3:27. 10.1186/s40168-015-0090-9 26113976 PMC4480905

[71] Gurnee EA Ndao IM Johnson JR: Gut Colonization of Healthy Children and Their Mothers With Pathogenic Ciprofloxacin-Resistant *Escherichia coli*. *J Infect Dis.* 2015;212(12):1862–8. 10.1093/infdis/jiv278 25969564 PMC4655851

[72] Gibson MK Wang B Ahmadi S: Developmental dynamics of the preterm infant gut microbiota and antibiotic resistome. *Nat Microbiol.* 2016;1:16024. 10.1038/nmicrobiol.2016.24 27572443 PMC5031140

[73] Gibson MK Crofts TS Dantas G: Antibiotics and the developing infant gut microbiota and resistome. *Curr Opin Microbiol.* 2015;27:51–6. 10.1016/j.mib.2015.07.007 26241507 PMC4659777

[74] Gasparrini AJ Crofts TS Gibson MK: Antibiotic perturbation of the preterm infant gut microbiome and resistome. *Gut Microbes.* 2016;7(5):443–9. 10.1080/19490976.2016.1218584 27472377 PMC5154371

[75] Centers for Disease Control and Prevention: National Listeria surveillance annual summary, 2014.2015 Reference Source

[76] Tappero JW Schuchat A Deaver KA: Reduction in the incidence of human listeriosis in the United States. Effectiveness of prevention efforts? The Listeriosis Study Group. *JAMA.* 1995;273(14):1118–22. 10.1001/jama.1995.03520380054035 7707600

[77] Lamont RF Sobel J Mazaki-Tovi S: Listeriosis in human pregnancy: a systematic review. *J Perinat Med.* 2011;39(3):227–36. 10.1515/JPM.2011.035 21517700 PMC3593057

[78] Jackson KA Iwamoto M Swerdlow D: Pregnancy-associated listeriosis. *Epidemiol Infect.* 2010;138(10):1503–9. 10.1017/S0950268810000294 20158931

[79] Shank FR Elliot EL Wachsmuth I: US position on *Listeria* monocytogenes in foods. *Food Control.* 1996;7(4–5):229–34. 10.1016/S0956-7135(96)00041-2

[80] Silk BJ Date KA Jackson KA: Invasive listeriosis in the Foodborne Diseases Active Surveillance Network (FoodNet), 2004–2009: further targeted prevention needed for higher-risk groups. *Clin Infect Dis.* 2012;54(Suppl 5):S396–404. 10.1093/cid/cis268 22572660

[81] Ventura SJ Abma JC Mosher WD: Estimated pregnancy rates by outcome for the United States, 1990–2004. *Natl Vital Stat Rep.* 2008;56(15):1–25, 28. 18578105

[82] Lee B Newland JG Jhaveri R: Reductions in neonatal listeriosis: "Collateral benefit" of Group B streptococcal prophylaxis? *J Infect.* 2016;72(3):317–23. 10.1016/j.jinf.2015.12.015 26772166

[83] Dando SJ Mackay-Sim A Norton R: Pathogens penetrating the central nervous system: infection pathways and the cellular and molecular mechanisms of invasion. *Clin Microbiol Rev.* 2014;27(4):691–726. 10.1128/CMR.00118-13 25278572 PMC4187632

[84] Kim KS: Pathogenesis of bacterial meningitis: from bacteraemia to neuronal injury. *Nat Rev Neurosci.* 2003;4(5):376–85. 10.1038/nrn1103 12728265

[85] Doran KS Fulde M Gratz N: Host-pathogen interactions in bacterial meningitis. *Acta Neuropathol.* 2016;131(2):185–209. 10.1007/s00401-015-1531-z 26744349 PMC4713723

[86] Liechti FD Grandgirard D Leib SL: Bacterial meningitis: insights into pathogenesis and evaluation of new treatment options: a perspective from experimental studies. *Future Microbiol.* 2015;10(7):1195–213. 10.2217/fmb.15.43 26119836

[87] Robinson J: Colonization and infection of the respiratory tract: What do we know? *Paediatr Child Health.* 2004;9(1):21–4. 19654976 10.1093/pch/9.1.21 PMC2719511

[88] Bosch AA Biesbroek G Trzcinski K: Viral and bacterial interactions in the upper respiratory tract. *PLoS Pathog.* 2013;9(1):e1003057. 10.1371/journal.ppat.1003057 23326226 PMC3542149

[89] Pericone CD Overweg K Hermans PW: Inhibitory and bactericidal effects of hydrogen peroxide production by *Streptococcus pneumoniae* on other inhabitants of the upper respiratory tract. *Infect Immun.* 2000;68(7):3990–7. 10.1128/IAI.68.7.3990-3997.2000 10858213 PMC101678

[90] Margolis E Yates A Levin BR: The ecology of nasal colonization of *Streptococcus pneumoniae, Haemophilus influenzae* and *Staphylococcus aureus*: the role of competition and interactions with host's immune response. *BMC Microbiol.* 2010;10:59. 10.1186/1471-2180-10-59 20178591 PMC2844402

[91] Lysenko ES Ratner AJ Nelson AL: The role of innate immune responses in the outcome of interspecies competition for colonization of mucosal surfaces. *PLoS Pathog.* 2005;1(1):e1. 10.1371/journal.ppat.0010001 16201010 PMC1238736

[92] Devine VT Jefferies JM Clarke SC: Nasopharyngeal Bacterial Carriage in the Conjugate Vaccine Era with a Focus on Pneumococci. *J Immunol Res.* 2015;2015: 394368. 10.1155/2015/394368 26351646 PMC4553195

[93] Ngo CC Massa HM Thornton RB: Predominant Bacteria Detected from the Middle Ear Fluid of Children Experiencing Otitis Media: A Systematic Review. *PLoS One.* 2016;11(3):e0150949. 10.1371/journal.pone.0150949 26953891 PMC4783106

[94] Dunne EM Smith-Vaughan HC Robins-Browne RM: Nasopharyngeal microbial interactions in the era of pneumococcal conjugate vaccination. *Vaccine.* 2013;31(19):2333–42. 10.1016/j.vaccine.2013.03.024 23523773

[95] de Steenhuijsen Piters WA Bogaert D: Unraveling the Molecular Mechanisms Underlying the Nasopharyngeal Bacterial Community Structure. *MBio.* 2016;7(1):e00009–16. 10.1128/mBio.00009-16 26838716 PMC4742699

[96] Ampofo K Bender J Sheng X: Seasonal invasive pneumococcal disease in children: role of preceding respiratory viral infection. *Pediatrics.* 2008;122(2):229–37. 10.1542/peds.2007-3192 18676537

[97] Sajjan U Wang Q Zhao Y: Rhinovirus disrupts the barrier function of polarized airway epithelial cells. *Am J Respir Crit Care Med.* 2008;178(12):1271–81. 10.1164/rccm.200801-136OC 18787220 PMC2599868

[98] Suzuki K Bakaletz LO: Synergistic effect of adenovirus type 1 and nontypeable Haemophilus influenzae in a chinchilla model of experimental otitis media. *Infect Immun.* 1994;62(5):1710–8. 8168932 10.1128/iai.62.5.1710-1718.1994 PMC186390

[99] Mueller JE Gessner BD: A hypothetical explanatory model for meningococcal meningitis in the African meningitis belt. *Int J Infect Dis.* 2010;14(7):e553–9. 10.1016/j.ijid.2009.08.013 20018546

[100] Bharti N Broutin H Grais RF: Spatial dynamics of meningococcal meningitis in Niger: observed patterns in comparison with measles. *Epidemiol Infect.* 2012;140(8):1356–65. 10.1017/S0950268811002032 22009033 PMC3846174

[101] Cohen AL McMorrow M Walaza S: Potential Impact of Co-Infections and Co-Morbidities Prevalent in Africa on Influenza Severity and Frequency: A Systematic Review. *PLoS One.* 2015;10(6):e0128580. 10.1371/journal.pone.0128580 26068416 PMC4466242

[102] Jansen AG Sanders EA VAN DER Ende A: Invasive pneumococcal and meningococcal disease: association with influenza virus and respiratory syncytial virus activity? *Epidemiol Infect.* 2008;136(11):1448–54. 10.1017/S0950268807000271 18211724 PMC2870742

[103] Talbot TR Poehling KA Hartert TV: Seasonality of invasive pneumococcal disease: temporal relation to documented influenza and respiratory syncytial viral circulation. *Am J Med.* 2005;118(3):285–91. 10.1016/j.amjmed.2004.09.016 15745727

[104] Kim KS: Mechanisms of microbial traversal of the blood-brain barrier. *Nat Rev Microbiol.* 2008;6(8):625–34. 10.1038/nrmicro1952 18604221 PMC5206914

[105] Sutherland TC Quattroni P Exley RM: Transcellular passage of *Neisseria meningitidis* across a polarized respiratory epithelium. *Infect Immun.* 2010;78(9):3832–47. 10.1128/IAI.01377-09 20584970 PMC2937448

[106] Spinosa MR Progida C Talà A: The *Neisseria meningitidis* capsule is important for intracellular survival in human cells. *Infect Immun.* 2007;75(7):3594–603. 10.1128/IAI.01945-06 17470547 PMC1932921

[107] Nikulin J Panzner U Frosch M: Intracellular survival and replication of *Neisseria meningitidis* in human brain microvascular endothelial cells. *Int J Med Microbiol.* 2006;296(8):553–8. 10.1016/j.ijmm.2006.06.006 17010667

[108] Magee AD Yother J: Requirement for capsule in colonization by *Streptococcus pneumoniae*. *Infect Immun.* 2001;69(6):3755–61. 10.1128/IAI.69.6.3755-3761.2001 11349040 PMC98386

[109] Nelson AL Roche AM Gould JM: Capsule enhances pneumococcal colonization by limiting mucus-mediated clearance. *Infect Immun.* 2007;75(1):83–90. 10.1128/IAI.01475-06 17088346 PMC1828419

[110] Weiser JN: The pneumococcus: why a commensal misbehaves. *J Mol Med (Berl).* 2010;88(2):97–102. 10.1007/s00109-009-0557-x 19898768 PMC4487619

[111] Turk DC: The pathogenicity of *Haemophilus influenzae*. *J Med Microbiol.* 1984;18(1):1–16. 10.1099/00222615-18-1-1 6146721

[112] Stephens DS Greenwood B Brandtzaeg P: Epidemic meningitis, meningococcaemia, and *Neisseria meningitidis*. *Lancet.* 2007;369(9580):2196–210. 10.1016/S0140-6736(07)61016-2 17604802

[113] Overturf GD: Indications for the immunological evaluation of patients with meningitis. *Clin Infect Dis.* 2003;36(2):189–94. 10.1086/345527 12522751

[114] Kim KS Itabashi H Gemski P: The K1 capsule is the critical determinant in the development of Escherichia coli meningitis in the rat. *J Clin Invest.* 1992;90(3):897–905. 10.1172/JCI115965 1326000 PMC329944

[115] Dietzman DE Fischer GW Schoenknecht FD: Neonatal Escherichia coli septicemia--bacterial counts in blood. *J Pediatr.* 1974;85(1):128–30. 10.1016/S0022-3476(74)80308-2 4604810

[116] Sullivan TD LaScolea LJ Jr Neter E: Relationship between the magnitude of bacteremia in children and the clinical disease. *Pediatrics.* 1982;69(6):699–702. 6804923

[117] Bell LM Alpert G Campos JM: Routine quantitative blood cultures in children with Haemophilus influenzae or Streptococcus pneumoniae bacteremia. *Pediatrics.* 1985;76(6):901–4. 3877910

[118] Fuchs E Untucht C Rohde M: Capsule Contributes to Transmigration of Streptococcus pneumoniae Serotype 7F Meningitis Isolates through Complex Blood Brain Barrier Models. *J Bacteriol Parasitol.* 2014;03 10.4172/2155-9597.1000142

[119] Baltas I Tsoulfa S Sakellariou P: Posttraumatic meningitis: bacteriology, hydrocephalus, and outcome. *Neurosurgery.* 1994;35(3):422–6; discussion 426-7. 10.1097/00006123-199409000-00009 7800133

[120] Ginsberg L Kidd D: Chronic and recurrent meningitis. *Pract Neurol.* 2008;8(6):348–61. 10.1136/jnnp.2008.157396 19015295

[121] van den Aardweg MT Rovers MM de Ru JA: A systematic review of diagnostic criteria for acute mastoiditis in children. *Otol Neurotol.* 2008;29(6):751–7. 10.1097/MAO.0b013e31817f736b 18617870

[122] Samuel J Fernandes CM Steinberg JL: Intracranial otogenic complications: a persisting problem. *Laryngoscope.* 1986;96(3):272–8. 10.1288/00005537-198603000-00007 3951303

[123] Younis RT Anand VK Childress C: Sinusitis complicated by meningitis: current management. *Laryngoscope.* 2001;111(8):1338–42. 10.1097/00005537-200108000-00006 11568566

[124] Simon TD Butler J Whitlock KB: Risk factors for first cerebrospinal fluid shunt infection: findings from a multi-center prospective cohort study. *J Pediatr.* 2014;164(6):1462–8.e2. 10.1016/j.jpeds.2014.02.013 24661340 PMC4035376

[125] Reefhuis J Honein MA Whitney CG: Risk of bacterial meningitis in children with cochlear implants. *N Engl J Med.* 2003;349(5):435–45. 10.1056/NEJMoa031101 12890842

[126] Brouwer MC de Gans J Heckenberg SG: Host genetic susceptibility to pneumococcal and meningococcal disease: a systematic review and meta-analysis. *Lancet Infect Dis.* 2009;9(1):31–44. 10.1016/S1473-3099(08)70261-5 19036641

[127] Kanneganti TD Lamkanfi M Núñez G: Intracellular NOD-like receptors in host defense and disease. *Immunity.* 2007;27(4):549–59. 10.1016/j.immuni.2007.10.002 17967410

[128] Vázquez-Boland JA Kuhn M Berche P: *Listeria* pathogenesis and molecular virulence determinants. *Clin Microbiol Rev.* 2001;14(3):584–640. 10.1128/CMR.14.3.584-640.2001 11432815 PMC88991

[129] Whiteside SA Razvi H Dave S: The microbiome of the urinary tract--a role beyond infection. *Nat Rev Urol.* 2015;12(2):81–90. 10.1038/nrurol.2014.361 25600098

[130] Wettergren B Jodal U: Spontaneous clearance of asymptomatic bacteriuria in infants. *Acta Paediatr Scand.* 1990;79(3):300–4. 10.1111/j.1651-2227.1990.tb11460.x 2333743

[131] Tebruegge M Pantazidou A Curtis N: Question 1. How common is co-existing meningitis in infants with urinary tract infection? *Arch Dis Child.* 2011;96(6):602–6. 10.1136/adc.2011.215277 21562337

[132] Carl MA Ndao IM Springman AC: Sepsis from the gut: the enteric habitat of bacteria that cause late-onset neonatal bloodstream infections. *Clin Infect Dis.* 2014;58(9):1211–8. 10.1093/cid/ciu084 24647013 PMC3982840

[133] Aloisio I Mazzola G Corvaglia LT: Influence of intrapartum antibiotic prophylaxis against group B *Streptococcus* on the early newborn gut composition and evaluation of the anti- *Streptococcus* activity of *Bifidobacterium* strains. *Appl Microbiol Biotechnol.* 2014;98(13):6051–60. 10.1007/s00253-014-5712-9 24687755

[134] Keski-Nisula L Kyynäräinen H Kärkkäinen U: Maternal intrapartum antibiotics and decreased vertical transmission of *Lactobacillus* to neonates during birth. *Acta Paediatr.* 2013;102(5):480–5. 10.1111/apa.12186 23398392

[135] Mazzola G Murphy K Ross RP: Early Gut Microbiota Perturbations Following Intrapartum Antibiotic Prophylaxis to Prevent Group B Streptococcal Disease. *PLoS One.* 2016;11(6):e0157527. 10.1371/journal.pone.0157527 27332552 PMC4917232

[136] Azad MB Konya T Persaud RR: Impact of maternal intrapartum antibiotics, method of birth and breastfeeding on gut microbiota during the first year of life: a prospective cohort study. *BJOG.* 2016;123(6):983–93. 10.1111/1471-0528.13601 26412384

[137] Lim ES Zhou Y Zhao G: Early life dynamics of the human gut virome and bacterial microbiome in infants. *Nat Med.* 2015;21(10):1228–34. 10.1038/nm.3950 26366711 PMC4710368

[138] La Rosa PS Warner BB Zhou Y: Patterned progression of bacterial populations in the premature infant gut. *Proc Natl Acad Sci U S A.* 2014;111(34):12522–7. 10.1073/pnas.1409497111 25114261 PMC4151715

[139] Norman JM Handley SA Virgin HW: Kingdom-agnostic metagenomics and the importance of complete characterization of enteric microbial communities. *Gastroenterology.* 2014;146(6):1459–69. 10.1053/j.gastro.2014.02.001 24508599 PMC4009354

[140] Maródi L: Neonatal innate immunity to infectious agents. *Infect Immun.* 2006;74(4):1999–2006. 10.1128/IAI.74.4.1999-2006.2006 16552028 PMC1418902

[141] Levy O: Innate immunity of the newborn: basic mechanisms and clinical correlates. *Nat Rev Immunol.* 2007;7(5):379–90. 10.1038/nri2075 17457344

[142] Ygberg S Nilsson A: The developing immune system - from foetus to toddler. *Acta Paediatr.* 2012;101(2):120–7. 10.1111/j.1651-2227.2011.02494.x 22003882

[143] Burchett SK Corey L Mohan KM: Diminished interferon-gamma and lymphocyte proliferation in neonatal and postpartum primary herpes simplex virus infection. *J Infect Dis.* 1992;165(5):813–8. 10.1093/infdis/165.5.813 1314868

[144] Sullender WM Miller JL Yasukawa LL: Humoral and cell-mediated immunity in neonates with herpes simplex virus infection. *J Infect Dis.* 1987;155(1):28–37. 10.1093/infdis/155.1.28 3025306

